# Rule-based definition of muscle bundles in patient-specific models of the left atrium

**DOI:** 10.3389/fphys.2022.912947

**Published:** 2022-10-12

**Authors:** Simone Rossi, Laryssa Abdala, Andrew Woodward, John P. Vavalle, Craig S. Henriquez, Boyce E. Griffith

**Affiliations:** ^1^ Department of Mathematics, UNC Chapel Hill, Chapel Hill, NC, United States; ^2^ Advanced Medical Imaging Lab, UNC Chapel Hill, Chapel Hill, NC, United States; ^3^ Department of Medicine, UNC Chapel Hill, Chapel Hill, NC, United States; ^4^ Department of Biomedical Engineering, Duke University, Durham, NC, United States; ^5^ Department of Biomedical Engineering, UNC Chapel Hill, Chapel Hill, NC, United States; ^6^ McAllister Heart Institute, UNC Chapel Hill, Chapel Hill, NC, United States

**Keywords:** left atrial fiber architecture, fiber reconstruction, rule-based model, electrophysiology simulations, finite element method

## Abstract

Atrial fibrillation (AF) is the most common arrhythmia encountered clinically, and as the population ages, its prevalence is increasing. Although the CHA_2_DS_2^−^_VASc score is the most used risk-stratification system for stroke risk in AF, it lacks personalization. Patient-specific computer models of the atria can facilitate personalized risk assessment and treatment planning. However, a challenge faced in creating such models is the complexity of the atrial muscle arrangement and its influence on the atrial fiber architecture. This work proposes a semi-automated rule-based algorithm to generate the local fiber orientation in the left atrium (LA). We use the solutions of several harmonic equations to decompose the LA anatomy into subregions. Solution gradients define a two-layer fiber field in each subregion. The robustness of our approach is demonstrated by recreating the fiber orientation on nine models of the LA obtained from AF patients who underwent WATCHMAN device implantation. This cohort of patients encompasses a variety of morphology variants of the left atrium, both in terms of the left atrial appendages (LAAs) and the number of pulmonary veins (PVs). We test the fiber construction algorithm by performing electrophysiology (EP) simulations. Furthermore, this study is the first to compare its results with other rule-based algorithms for the LA fiber architecture definition available in the literature. This analysis suggests that a multi-layer fiber architecture is important to capture complex electrical activation patterns. A notable advantage of our approach is the ability to reconstruct the main LA fiber bundles in a variety of morphologies while solving for a small number of harmonic fields, leading to a comparatively straightforward and reproducible approach.

## 1 Introduction

In recent years, computational models have been increasingly used to investigate atrial cardiac electrophysiology, mechanics, and blood flow. These models have been used for a variety of purposes, including to understand the mechanisms underlying atrial fibrillation and to predict the risk of thromboembolism (Zhang and Gay, 2008; Krummen et al., 2012; Chang et al., 2014; Masci et al., 2017; Bosi et al., 2018; Aronis et al., 2019). Atrial fibrillation is the most common electrical dysfunction of the heart encountered clinically (Korantzopoulos et al., 2018; Patel et al., 2018; Bhat et al., 2020; Lippi et al., 2021). Under this condition, the activation of the heart muscle is disordered, with fibers or groups of fibers contracting independently, and normal systole and diastole no longer occur (Garrey, 1924). The main clinical concerns for patients with atrial fibrillation is thromboembolic stroke (Jørgensen et al., 1996; Lin et al., 1996; Go et al., 2001): abnormal blood flow associated with asynchronous atrial contraction creates the conditions for blood clots to form in the left atrial appendage (Jame and Barnes, 2020). With an aging population, in both the European Union (Krijthe et al., 2013) and the United States (Chung et al., 2020), the number of patients with atrial fibrillation is projected to more than double in the next 30 years. To reduce the risk of thromboembolic events, oral anticoagulant therapy is the preferred treatment for atrial fibrillation patients (January et al., 2019; Jame and Barnes, 2020; Hindricks et al., 2021) with a high CHA_2_DS_2^−^_VASc score (Friberg et al., 2012). The CHA_2_DS_2^−^_VASc score is the most commonly used risk-stratification scoring system to guide atrial fibrillation treatment, although other approaches have been proposed (Borre et al., 2018; Jame and Barnes, 2020). Because all currently used risk-stratification schemes lack personalization, many researchers have created patient-specific computer simulations of atrial fibrillation (Zhang and Gay, 2008; Bosi et al., 2018; García-Isla et al., 2018; Masci et al., 2020; Morales Ferez et al., 2021). An effective alternative to treatment for patients not eligible for anticoagulant therapy is the placement of an occlusion device in the left atrial appendage (Kaafarani et al., 2020). The WATCHMAN device is a commercially available occlusion device that prevents large thrombi from exiting the left atrial appendage preventing stroke events. This approach has demonstrated an equivalent reduction in stroke and mortality compared to warfarin in patients with atrial fibrillation (Chanda and Reilly, 2017; Safavi-Naeini and Rasekh, 2018).

One of the challenges faced in the creation of patient-specific atrial models is the complexity of the atrial muscle arrangement (Krueger et al., 2011; Aronis et al., 2019). This intricate architecture has been highlighted by multiple studies since the early 1800s (Gerdy, 1823; Keith, 1903; Tandler, 1913; Papez, 1920; Thomas, 1959; Wang et al., 1995; Ho et al., 1999, 2012). Indeed, the atrial muscle is composed of several muscle bundles overlapping and crossing each other (Ho et al., 2002; Ho and Sanchez-Quintana, 2009; Ho et al., 2012; Pashakhanloo et al., 2016). Additionally, structural remodeling and fibrosis in patients with atrial fibrillation can drastically change the local orientation of the muscle fibers in unpredictable ways (Pashakhanloo et al., 2016; Aronis et al., 2019). While early computational studies of the atria circumvented the problem of defining muscle fiber directions by assuming isotropic material properties (Lorange and Gulrajani, 1993; Zhang et al., 2003; Pandit et al., 2005; Dokos et al., 2007), it is increasingly common to subdivide the atria into regions, with each region associated to a different fiber orientation (Harrild and Henriquez, 2000; Vigmond et al., 2001; Jacquemet et al., 2003; Seemann et al., 2006; Gonzales et al., 2013; Tobón et al., 2013; Ferrer et al., 2015; Moyer et al., 2015; Wachter et al., 2015; Fastl et al., 2018; Piersanti et al., 2021; Zheng et al., 2021).

Many computational studies have included information of the fiber fields, and several algorithms have been published for generating anisotropy information in patient specific models. These algorithms can be divided in two main categories: atlas-based (McDowell et al., 2012; Satriano et al., 2013; Labarthe et al., 2014; Roney et al., 2018; Hoermann et al., 2019; Roney et al., 2021) and rule-based (Hermosillo, 2008; Krueger et al., 2011; Labarthe et al., 2012; Fastl et al., 2018; Saliani et al., 2019; Piersanti et al., 2021). Atlas-based atrial fiber construction is based on creating a mapping between a patient geometry of the left atrium and a simplified atlas geometry. Once the mapping is built, the muscle fiber orientation from the atlas geometry is transferred directly onto the new geometry. There are two major challenges with this approach: atrial anatomy is highly variable, and different subjects have different appendage morphologies and numbers of pulmonary veins; the data to create the atlas is limited and proprietary. Rule-based approaches include in-painting methods, in which the curves representing the main muscle bundles are drawn manually on the atrium, (Labarthe et al., 2012; Saliani et al., 2019), whereas others involve complex algorithmic subdivision of the atrial geometry (Hermosillo, 2008; Krueger et al., 2011). Recent rule-based algorithms use partial differential equations. In these algorithms, the fiber fields, and sometimes also the regions, are determined by solving Poisson-type equations with various boundary conditions. The gradients of the resulting harmonic fields form a local frame of reference that defines the local muscle fiber orientation. This approach generates fields that conform to each geometry and can be easily extended to consider various changes in the left atrial geometries. To our knowledge, only two algorithms of this type have been previously described for generating fibers in the left atrium: one developed by Fastl et al. (2018) and the other developed by Piersanti et al. (2021). The approach used by Fastl et al. (2018) subdivides the left atrial endocardium and epicardium in 151 regions after transferring predefined landmarks from an average atrial geometry. Each region is then associated to an harmonic field, whose gradient correspond to the local fiber direction. Given the challenges in implementing such an intricate method, Piersanti et al. (2021) proposed a simpler approach in which only four harmonic fields are computed on the patient-specific geometry. Although this algorithm captures some of the atrial fibers features correctly, it also misses some details. Further, it does not allow for the definition of different endocardial and epicardial fiber orientations.

Here, we propose an algorithm to create the fibers for the left atrium capable of approximating the complex method of Fastl et al. (2018) using only seven harmonic fields. The method requires the definition of common boundary sets (endocardium, epicardium, mitral valve ring, pulmonary veins) and two landmark points, one for the left atrial appendage and one for the fossa ovalis. The algorithm is semi-automated in the sense that, after solving for the harmonic fields, it is necessary only to set the thresholds that define the various subregions of the left atrium. We demonstrate the robustness of the proposed algorithm by recreating the fiber fields for patients who underwent WATCHMAN device implantation.

When describing our approach in this paper, the word ‘fiber’ is used to refer to a group of similarly oriented myocytes; such collections of myocytes are large enough to be seen by the naked eye (Ho et al., 2002). We use the word ‘bundle’ to indicate a collection of fibers with approximately equal alignment. We refer to overlapping bundles through the transmural direction as ‘layers’. We say the fibers are longitudinal if they are roughly perpendicular to the mitral valve orifice, and that they are circumferential if they run parallel to the mitral valve annulus (Sánchez-Quintana et al., 2014).

## 2 Materials and methods

### 2.1 Preprocessing of patient-specific anatomies

**FIGURE 1:**
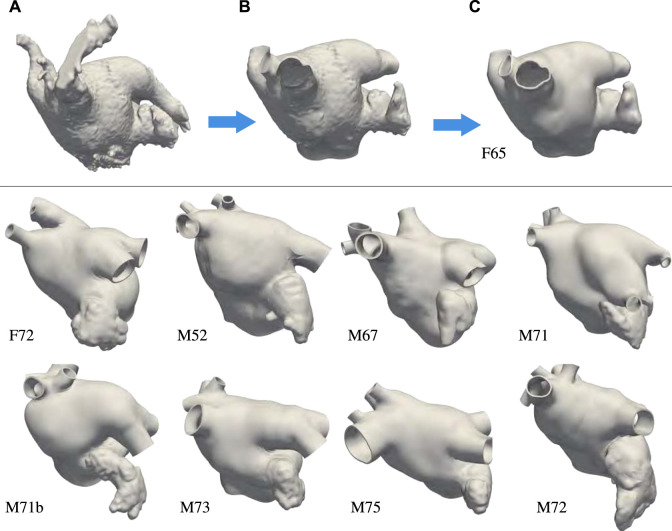
Top: Preprocessing steps: **(A)** Segmentation extracted from the patient computed tomography images. **(B)** Preprocessing of the endocardial surface after chopping the pulmonary veins opens and opening the mitral valve ring. **(C)** Thickened and retopologized atrial surface. Bottom: final preprocessed left atrial surfaces of eight patients who underwent WATCHMAN device implantation. We remark that most of these patients have accessory right pulmonary veins and two of them a common left pulmonary trunk. We label the anatomies using F and M, to indicate patients’ gender, followed by their age.

Nine left atrial anatomies of patients with AF were acquired at UNC Medical Center. All patients underwent a procedure to occlude their left atrial appendage (LAA) via WATCHMAN device implantation. The patient ages range from 52 to 75 years old. Two are female and seven are male. Patient data were de-identified and accessed using methods approved by UNC Institutional Review Board (under IRB protocol 18-0754). These patient-specific atrial morphologies have various pulmonary vein (PV) configurations, including PV anomalies often observed in atrial fibrillation patients (Marom et al., 2004; Kaseno et al., 2008), such as left common PV trunk, right middle lobe PV. Figure 1 shows the preprocessing for one of the patients and the final geometrical models of the left atrium (LA) of all the other patients.

The preprocessing of the atrial geometry was performed manually to preserve the anatomical features of each of the patients. Semi-automatic segmentation was performed using Intuition (TeraRecon) as illustrated in Figure 1 panels A–C. After segmentation, the endocardial atrial surface was preprocessed and extruded in MeshMixer (Autodesk), retopologized in Blender using the QuadRemesher (Exoside) plugin, and meshed via GMSH (Geuzaine and Remacle, 2009) to obtain a tetrahedral representation of the LA. Specifically, from the initial segmentation shown in Figure 1A, we reduced the number of elements reproducing the endocardial surface to about 50,000. After cutting, repairing, and smoothing parts of the PVs, the closed mitral valve (MV) opening was smoothed and removed from the surface. We separated the LAA surface when obvious self-contact of the lobes or contact with the PVs or the anterior wall were apparent. The LAA, MV ring, and pulmonary veins were smoothed, and their surfaces were remeshed with a target mesh size of 0.75 mm. Other unwanted features of the LA were removed and the surface was smoothed. Figure 1B shows the preprocessed endocardial surface. An initial epicardial surface was then created offsetting by 1 mm the endocardial surface in the outward normal direction. The epicardial LA surface without the LAA and the PVs was extruded by 1 mm in the normal direction again to obtain a total 2 mm atrial thickness (Beinart et al., 2011). After smoothing, the connected epicardial-endocardial surface was retopologized using quadrilateral elements with a target of about 10,000 points. From the quadrilateral surface mesh, we generated a tetrahedral mesh and marked the boundaries with physical groups to assign boundary conditions. Different boundary sets are assigned to the endocardium, epicardium, mitral valve ring, and each of the pulmonary veins. The tetrahedral mesh was uniformly refined three times to obtain at least 8 elements through the thickness. This choice was dictated by the necessity of a small mesh grid size for running electrophysiology simulations with linear finite elements Niederer et al. (2011); Pezzuto et al. (2016); Quarteroni et al. (2017). The resulting meshes have between 22,763,520 and 33,878,016 elements. Because of the uniform refinement, the quality of the initial mesh is preserved in the nested refinement. Figure 1C shows the final preprocessed geometry. Finally, we applied a rigid motion to all the geometries to align the MV ring with the *x* − *y* plane at *z* = 0. For someone who is skilled in the art of manipulating anatomies, the manual process takes approximately 30 min to complete depending on the quality of the segmentation.

### 2.2 Muscle fibers algorithm on a particular left atrial anatomy

We now describe the algorithm for generating the fiber architecture on the M75 left atrial anatomy shown in Figure 1, which has six PVs. Specifically, the right inferior and superior PVs branch out into two PVs each, resulting in a total of six ostia.

To assign the muscle fibers orientation in a patient-specific anatomy, we first solve for seven harmonic fields, *ϕ*_*i*_, *i* = 0, 1, 2, …, 6. Each field can be interpreted geometrically, as follows. The field *ϕ*_0_ represents the transmural distance between the endocardium and the epicardium. It is used to separate the epicardial and the endocardial layers and to define the transmural direction *s*. The field *ϕ*_1_ represents the distance between the fossa ovalis (FO) and the LAA. Given the field *ϕ*_1_, the user can select thresholds to define the FO and the LAA. The field *ϕ*_2_ sweeps the geometry in the lateral-septal direction, representing the distance between the left PVs and the right PVs. The field *ϕ*_3_ attains its smallest values on the PVs close to the posterior, achieving its maximum values on the PVs by the anterior wall. This field is used to identify the anterior and posterior walls. The field *ϕ*_4_ represents the distance from the MV ring to the PVs. Note that we constrained the field *ϕ*_4_ to be identically one within the LAA and identically zero within the FO. Because this anatomy has six PVs, the boundary conditions on the right PVs for *ϕ*_3_ and *ϕ*_4_ are adjusted to represent their intended geometric interpretations accordingly. The last two harmonic fields, *ϕ*_5_ and *ϕ*_6_, are more complicated because they represent interconnected distances between various regions of the LA. Specifically, *ϕ*_5_ connects the right PVs to the left PVs, the MV ring, and LAA. It is used to identify the right and left antras. Lastly, the field *ϕ*_6_ is similar to *ϕ*_2_, with an added boundary condition constraint in the MV ring. The MV ring is defined to be half-way between the right and left PVs. This last field aids in describing the fibers on the anterior wall.

**FIGURE 2:**
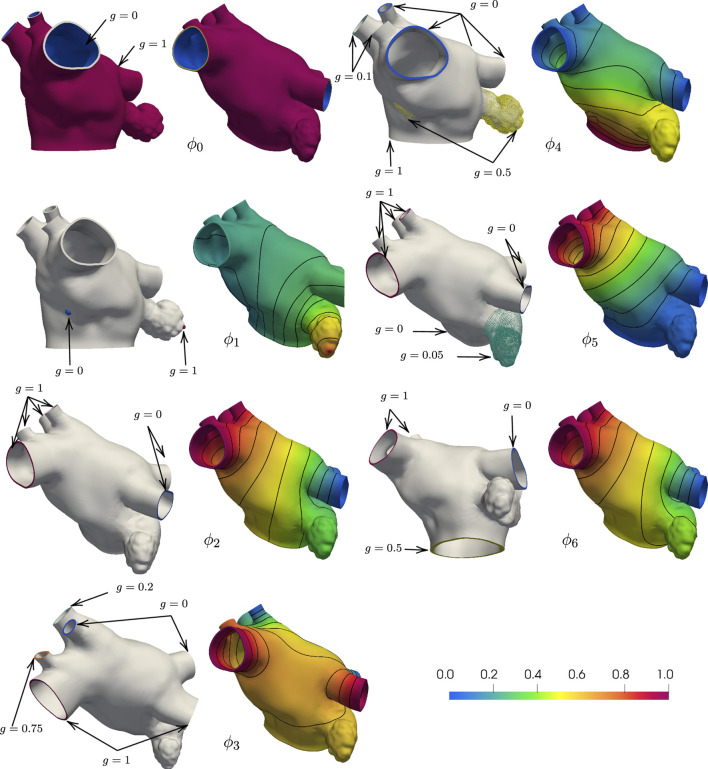
Boundary conditions and corresponding harmonic fields for M75. The field *ϕ*_0_ captures the distance from the endocardial and epicardial surfaces, respectively *g* = 0 and *g* = 1, and its gradient defines the transmural direction ***s***. Imposing *g* = 0 at the tip of the LAA and *g* = 1 at the center of the FO, the field *ϕ*_1_ sweeps from the LAA to the FO. Setting *g* = 0 on the left PVs and *g* = 1 on the right PVs, the field *ϕ*_2_ define the fibers on the anterior and posterior walls. With *g* = 1 on the superior PVs and *g* = 0 on the inferior PVs and intermediate values on the auxilliary PVs, the field *ϕ*_3_ separates the anterior and posterior walls. The field *ϕ*_4_ defines the Bachmann’s bundle, imposing *g* = 1 on the MV ring, *g* = 0.5 in the LAA and FO regions, and *g* = 0 on the major PVs and *g* = 0.1 on the right middle PVs. The field *ϕ*_5_ identifies the left and right antras setting *g* = 0 on the left PVs and MV ring, *g* = 0.5 on the LAA, and *g* = 1 on the right PVs. The field *ϕ*_6_ defines the fiber direction in the anterior wall, setting *g* = 0 on the left PVs, right inferior PV, and around the MV ring, *g* = 0.05 on the LAA, and *g* = 1 on the right PVs.

Each of these harmonic fields constructed as a solution to the partial differential equation Δ*ϕ*_*i*_ = 0, in Ω, with *ϕ*_*i*_ = *g*, on Γ_D_, and *∂ϕ*_*i*_/*∂n* = 0, on *∂*Ω\Γ_D_. Here, Ω is the anatomical domain, *∂*Ω is the boundary of Ω, and Γ_D_ is a notation for landmark points and boundary sets, which are used to impose Dirichlet boundary conditions. We remark that all meshes derived from the anatomies are pre-processed to contain boundary sets on the PVs and MV rims. In addition, landmark points on the LAA tip and in the center of the fossa ovalis (FO) are used to impose Dirichlet boundary conditions for field *ϕ*_1_. We show in Figure 2 the boundary data and the resulting harmonic fields for M75.

Once these harmonic fields have been determined, we split the LA into subregions that are inspired by studies on atrial anatomy (Ho and Sanchez-Quintana, 2009; Ho et al., 2012; Sánchez-Quintana et al., 2014). Although not explicitly defined as an output of the implemented algorithm, the subregions of the LA are used to assign fiber orientations. We show the subregions Ω_*k*_ ⊂ Ω, *k* ∈ {1, *…*, 17}, to explain how we set up the fiber directions in the LA. Mathematically, for a subregion Ω_*k*_ ⊂ Ω and each field *ϕ_i_*, we define two threshold parameters *α_i,k_* and *β_i,k_* and the corresponding set $A_{i,k}=\left\{x\in \Omega :\alpha_{i,k}<\phi_{i}\left(x\right)<\beta_{i,k}\right\}$ . The characteristic function of *A*_*i,k*_ is

$$\chi_{A_{i,k}}\left(x\right)=\left\{\begin{array}{cc} 1,\; & ifx\in A_{i,k},\\ 0,\; & otherwise. \end{array}\right.$$ (1)

**FIGURE 3:**
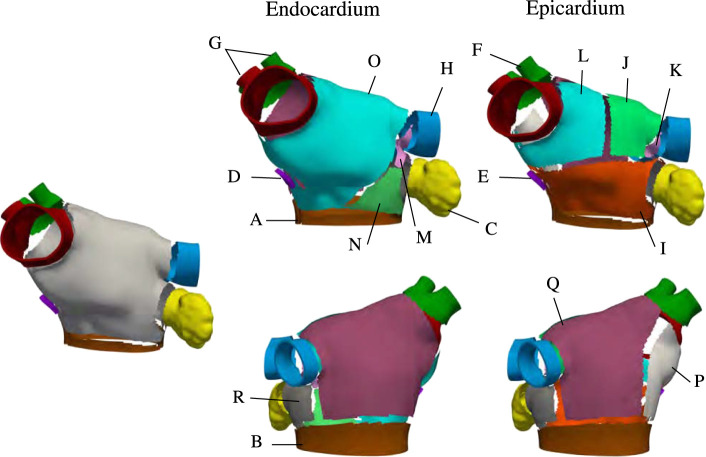
Region selection in M75. The left panel shows the regions common to the endocardial and epicardial layers that are first extracted by the algorithm After those are defined, the endocardial anterior is split into three regions and the epicardial anterior wall is split into four regions. Then, the lateral and posterior walls are defined in both layers. Notice that, in the epicardium, the inferior part of the septum is defined as separate subregion.

Each anatomical region Ω_*k*_ is defined by the characteristic function $r_{k}=\omega_{k}\;\prod_{i\in \left\{1,\ldots ,7\right\}}\chi_{A_{i,k}}\left(x\right)$ , in which $\omega_{k}=\Pi_{j=1}^{k-1}\left(1-r_{j}\right)$ , and *r*_0_ ≡ 0. The thresholds *α_i,k_*, *β_i,k_* are anatomy-dependent. This construction is such that, once a region is defined, it gets subtracted from the domain. Figure 3 shows the final decomposition on anatomy M75, and Table 1 displays the name of each of the subregions.

**TABLE 1:** Description of subregions shown in Figure 3.

Region label	Anatomical region	Layer	Fiber direction
A	Anterior floor	Epi/Endo	Circumferential
B	Posterior floor	Epi/Endo	Circumferential
C	Appendage	Epi/Endo	Circumferential
D	Fossa Ovalis	Endo	Circumferential
E	Fossa Ovalis	Epi	Longitudinal
F	Right inferior PV	Epi	Longitudinal
G	Right PVs	Epi/Endo	Circumferential
H	Left PVs	Epi/Endo	Circumferential
I	Bachmann’s Bundle	Epi	Circumferential
J	Left roof	Epi	Oblique
K	Left lateral ridge	Epi	Circumferential
L	Right roof	Epi	Longitudinal
M	Left lateral ridge	Endo	Circumferential
N	Anterior-lateral	Endo	Circumferential
O	Anterior wall	Endo	Oblique
P	Posterior-Septum	Epi	Longitudinal
Q	Posterior Wall	Epi/Endo	Longitudinal
R	Lateral Wall	Epi/Endo	Circumferential

The proposed algorithm creates two layers of muscle fibers–endocardial and epicardial–representing a simplification of the actual multi-layer LA architecture (Nathan and Eliakim, 1966; Wang et al., 1995; Ho et al., 2002, 2012; Pashakhanloo et al., 2016). Mathematically, this structure is represented by the vector field ***f*** defined over the domain Ω = ⋃_*k*_Ω_*k*_. More specifically, for each point in Ω_*k*_, we define a local frame of reference {***f***_*k*_, ***s***_*k*_, ***n***_*k*_}, in which the subscript *k* indicates the specific subregion Ω_*k*_. That is, ***f***_*k*_ is the restriction of *f* to Ω_*k*_, or $f_{k}={\left.f\right|}_{\Omega_{k}}$ . The local frame of reference {***f***_*k*_, ***s***_*k*_, ***n***_*k*_} is calculated using the gradients of the previously defined harmonic fields. Denoting with ***g***_*i*_ = ∇*ϕ*_*i*_, we define ***s*** = ***g***_0_ = ∇*ϕ*_0_. In other words, the vector ***s*** represents the transmural direction and its evaluation does not depend on the subregion. On the contrary, depending on the subregion Ω_*k*_, we define either ***f***_*k*_ = ***g***_*i*_ and ***n***_*k*_ = ***f***_*k*_ ×*s*_*k*_ or ***n***_*k*_ = ***g***_*i*_ and ***f***_*k*_ = ***n***_*k*_ × ***s***_*k*_. The step-by-step procedure used to create the local frames of reference is detailed in Algorithm 1.

**Table:** 

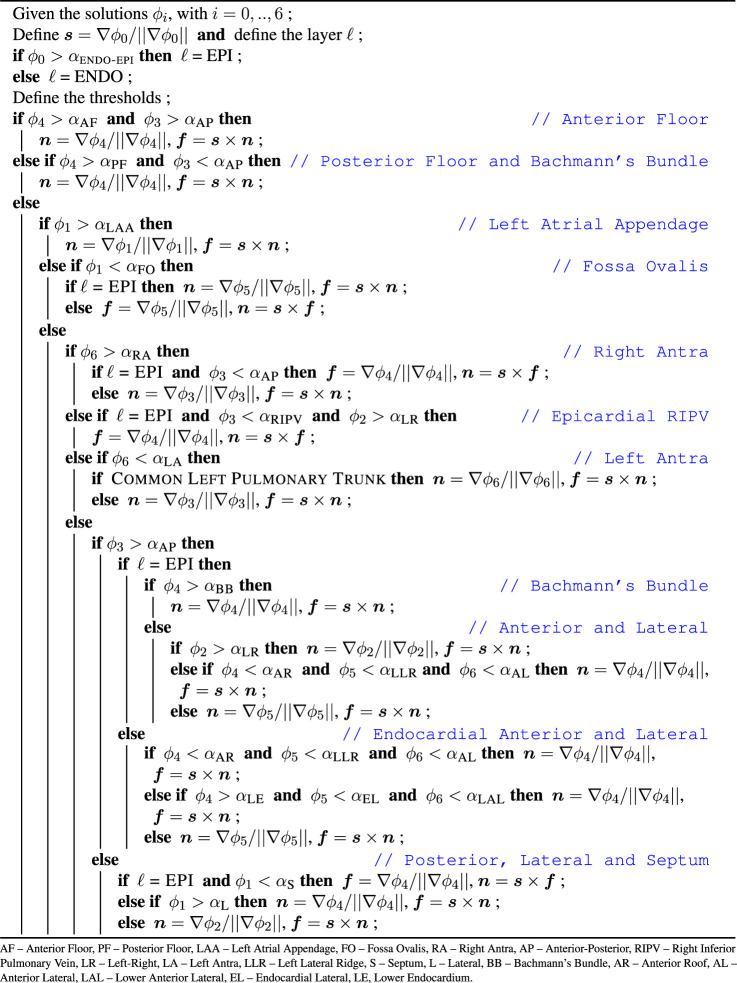				

ALGORITHM 1 Fiber fields generation–generates the orthonormal frame of reference {***f***, ***s***, ***n***}.

We defined the fiber structure to reproduce the model by Fastl et al. (2018) as closely as possible using the gradients of the seven harmonic fields. This architecture is adjusted for geometries with various pulmonary vein configurations. The fiber direction on the floor regions (A, B), and on the appendage (C) are assumed to run circumferentially along the mitral valve rings and the ostium of the appendage (Ho et al., 2012). In the fossa ovalis, the complex fibers architecture is simplified assuming longitudinal and circumferential arrangements in the epicardial (E) and endocardial (D) layer, respectively. In the left pulmonary veins and the right superior pulmonary veins and the right superior accessory pulmonary veins (F, G, H), the fiber are assumed circumferential. In the right inferior pulmonary vein and the possibly nearby right accessory pulmonary veins the fibers run longitudinally and circumferentially, in the epicardial (F) and endocardial (G) layer, respectively. The latter pattern is also defined on the posterior-septal region (P). The septoatrial bundle, descending obliquely into the bottom part of septal wall near the mitral valve, define the endocardial layer of the anterior wall (N, O). Another major fascicle of the septoatrial bundle combines into the longitudinal fibers of the posterior wall (Q). According to Pashakhanloo et al. (2016), these bundles are extensions of the fiber tracts from the roof and lateral wall. The epicardial layer of anterior wall is mainly composed by the septoatrial bundle (J, L) and the Bachmann’s bundle (I). The septoatrial bundle extends longitudinally onto the posterior wall (Q). The fibers from the lateral wall cross over the septopulmonary bundle (Pashakhanloo et al., 2016) on the posterior floor (B). The lateral wall (R) has leftward fibers that are part of the septoatrial bundle on the endocardial layer. In the left lateral ridge (K, M), the region between the left pulmonary veins and the mouth of the left atrial appendage, runs the oblique vein of Marshall (Sánchez-Quintana et al., 2014), strands of the septopulmonary and septoatrial bundles (Cabrera et al., 2008), resulting in predominantly circumferentially aligned strands (Cabrera et al., 2008; Ho et al., 2012; Sánchez-Quintana et al., 2014). This region also comprises myofibers from the extension of the Bachmann’s bundle, which is the most prominent bundle on the epicardial layer (Cabrera et al., 2008).

**FIGURE 4:**
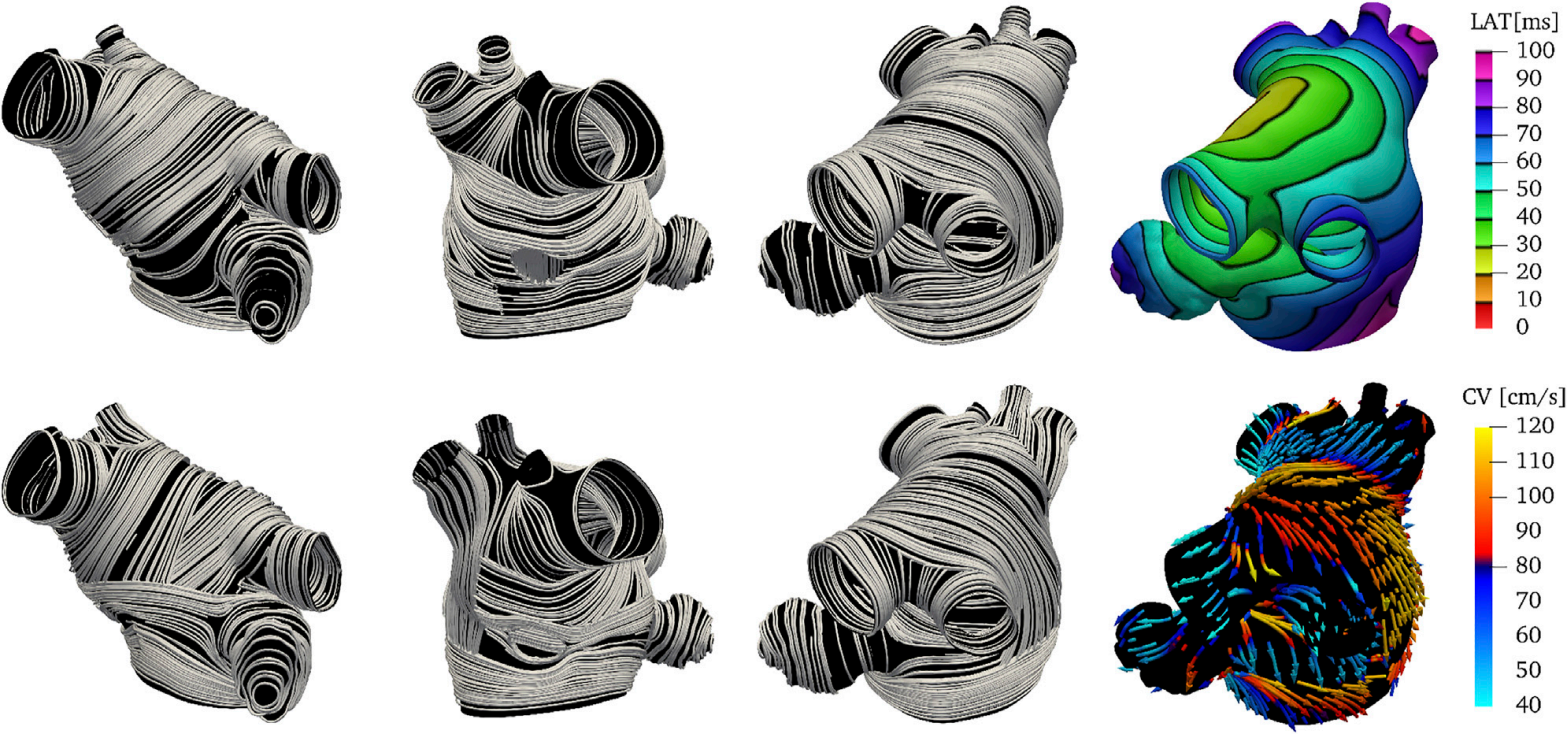
Endocardial (top) and epicardial (bottom) fiber fields, activation times and conduction velocities on M75. The Bachmann’s bundle on the epicardial layer wraps around the left atrial appendage. Note that the orientation of the fibers in the left and right carina and in the left lateral ridge are an improvement over existing methods in representing the anatomical muscle bundles. Similarly to Fastl et al. (2018), the fibers in the right posterior pulmonary veins are set longitudinally instead of circumferentially. The activation times and the conduction velocities show two main paths of activations: one circumferential through the Bachmann’s bundle and another superior and then posterior through the septoatrial bundle.

To test the generated fiber field, we perform electrophisiology simulations using the monodomain equation in which we impose an initial stimulus at the top of Bachmann’s Bundle (BB) at the center of the anterior wall. Figure 4 shows the resulting fiber field, local activation times (LAT), and conduction velocity (CV). The fiber orientation in the left and right carina and in the left lateral ridge are an improvement over existing rule-based methods in representing the anatomical muscle bundles.

### 2.3 Common left pulmonary trunk

Several imaging studies have found substantial variation in PV anatomy among patients with AF (Wittkampf et al., 2003; Schwartzman et al., 2004; Mlčochová et al., 2005; Cronin et al., 2007; Kaseno et al., 2008; Bittner et al., 2011). The left common PV has been found to be a prevalent anomaly among patients with AF (Porres et al., 2013; McLellan et al., 2014; Chen et al., 2017; Stabile et al., 2017). Here we describe how the method detailed in Subsection 2.2 can be extended to anatomies with left common pulmonary trunk. We only highlight the changes to the algorithm needed to recreate the fiber fields on anatomy M52, which has five right PVs and a left common pulmonary trunk.

**FIGURE 5:**
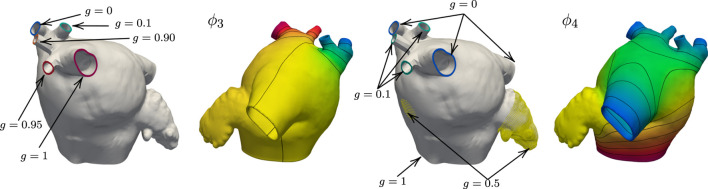
Boundary data and harmonic solutions on M52. We set *g* = 0 on the right inferior PV and *g* = 1 right superior PV when solving for *ϕ*_3_. The accessory PVs are set to intermediate values between 0 and 1. On the left common pulmonary trunk we impose natural boundary conditions. This field is used to define the anterior and posterior walls. The boundary conditions for *ϕ*_4_ are *g* = 0 on the right superior, inferior, and left common PVs. The value of *g* on the three right PVs that lie between the superior-most and inferior-most is 0.1. The boundary condition on the MV ring is *g* = 1, and the LAA and FO regions are set to 0.5.

**FIGURE 6:**
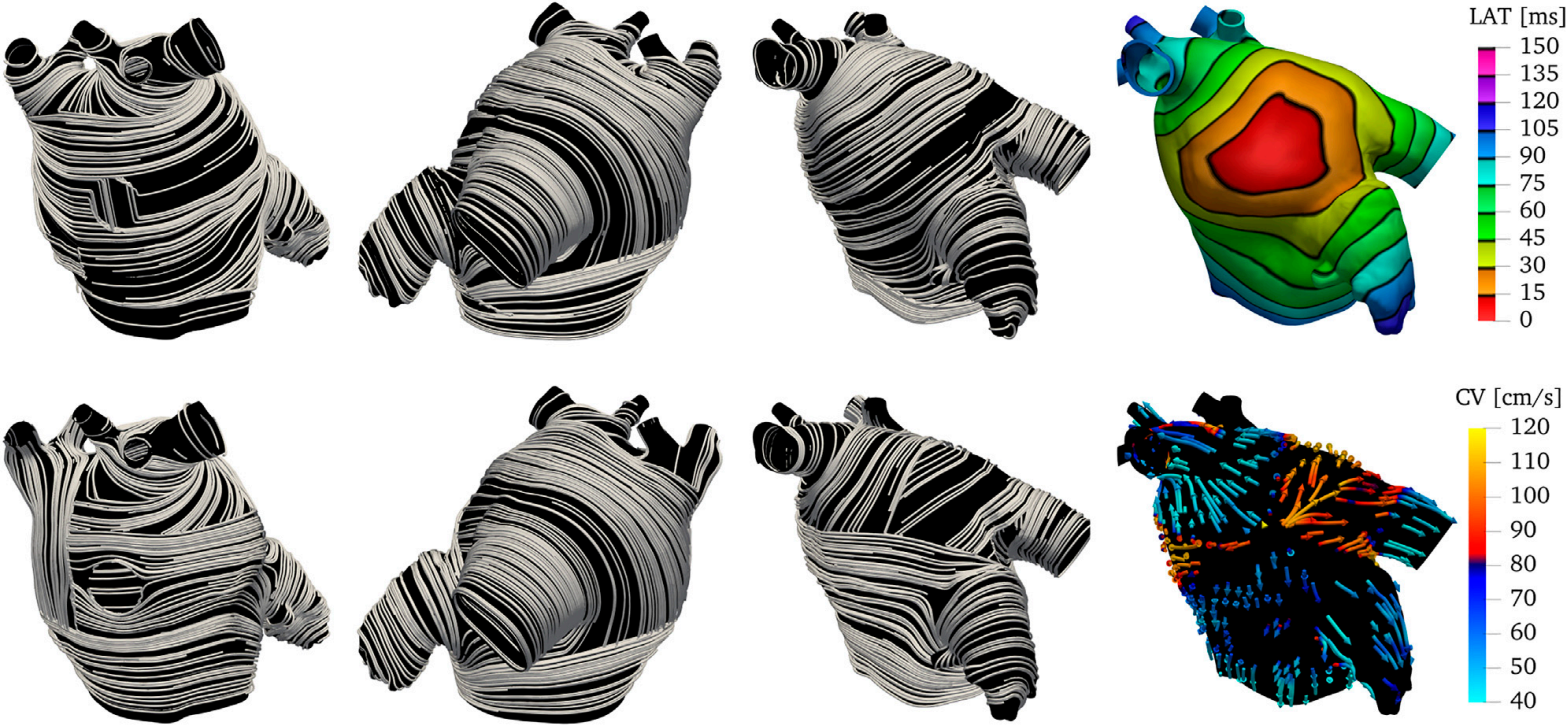
Endocardial (top) and epicardial (bottom) fiber fields, activation times and conduction velocities on M52. The presence of the left common pulmonary trunk is easily accounted by the algorithm. The Bachmann’s bundle on the epicardial layer wraps around the left atrial appendage. Note that the orientation of the fibers in the right carina and in the left lateral ridge are an improvement over existing methods in representing the anatomical muscle bundles. Similarly to Fastl et al. (2018) the fibers in the right posterior pulmonary veins are set longitudinally instead of circumferentially. The activation times and the conduction velocities show two main path of activations: one circumferential through the Bachmann’s bundle and another superior and then posterior through the septoatrial bundle.

We maintain the same geometrical interpretations of the harmonic fields as in the previous example. However, we adjust the boundary conditions for *ϕ*_3_ and *ϕ*_4_ to preserve their interpretation. In this case, we use natural homogeneous boundary conditions for *ϕ*_3_ on the left common pulmonary trunk. This modification is done so the anterior and posterior walls can be separated using such a field. For the field *ϕ*_4_, we set the three right PVs that lie in between the superior-most and inferior-most PVs to 0.1. Figure 5 shows the changes in the boundary conditions and resulting fields *ϕ*_3_ and *ϕ*_4_ on M52. Besides the changes in boundary conditions, we alter the logic in Algorithm 1 to consider a common left pulmonary trunk as a single pulmonary vein without the left carina. The local frame of reference on the left common PV region is calculated by setting ***n*** = ***g***_6_ = ∇*ϕ*_6_, opposed to ***n*** = ***g***_3_ = ∇*ϕ*_3_ used to define the fibers on the left PVs on the M75 anatomy. Figure 6 shows the resulting fiber architecture, activation times, and conduction velocities on M52.

### 2.4 Mathematical and numerical methods

We analyzed the influence of the proposed algorithm on the estimated local activation times and propagation patterns in various atrial morphologies by solving the anisotropic monodomain equation. The local fiber orientation is introduced in the conductivity tensor as ***σ*** = *σ* ^f^ ***f*** ⊗ ***f*** + *σ* ^s^ ***s*** ⊗ ***s*** + *σ* ^n^ ***n*** ⊗ ***n***, in which the set {***f***, ***s***, ***n***} represents the local frame of reference. The field ***f*** is parallel to the longitudinal orientation of the cardiac cells, ***s*** represents the transmural direction and ***n*** = ***f*** × ***s*** is the orthogonal transversal direction. The parameters *σ* ^f^, *σ* ^s^, and *σ* ^n^ are the conductivity coefficients in the ***f***, ***s***, and ***n*** directions, respectively. The monodomain equations expressed in terms of the transmembrane potential difference *V* are

$$\begin{array}{ll} \chi \left(C_{m}\frac{\partial V}{\partial t}+I_{ion}\left(y,V\right)+I_{stimulus}\left(t\right)\right) & =\nabla \cdot \left(\sigma \nabla V\right),\\ \frac{\partial y}{\partial t} & =g\left(y,V\right), \end{array}$$ (2)

in which *χ* is the membrane area per unit tissue volume, *I*_ion_ is transmembrane current density, and *C*_m_ is the specific membrane capacitance per unit membrane area. The transmembrane current *I*_ion_ is a function of a set of state variables ***y***, representing gating variables and other state variables, possibly including local ionic concentrations. The dynamics of the state variable ***y*** are described by a system of ordinary differential equations. Herein, we characterize *I*_ion_, ***y***, and ***g*** (***y***, *V*) using the Cherry-Ehrlich-Nattel-Fenton human atrial ionic model (Cherry et al., 2007). The parameters used in the numerical simulations are reported in Table 2.

**TABLE 2:** Parameters used in the monodomain model described by Eq. (2).

$\chi \;\left[{cm}^{-1}\right]$	$C_{m}\;\left[\mu {Fcm}^{-2}\right]$	$\sigma^{f}\;\left[{mScm}^{-1}\right]$	$\sigma^{s}\;\left[{mScm}^{-1}\right]$	$\sigma^{n}\;\left[{mScm}^{-1}\right]$	$I_{stimulus}\;\left[\mu {Acm}^{-3}\right]$
1000	1	1.3342	0.36	0.36	1000 if *t* < 2 ms

The Laplace problems and the monodomain model were discretized in space using linear tetrahedral finite elements. The transmembrane voltage was approximated using the IMEX temporal scheme SBDF1 (Ascher et al., 1995). Briefly, we first solved for the gating variables ***y***, then we used their updated values to evaluate the ionic current *I*_ion_, which was treated explicitly. The diffusion term in the monodomain model was treated implicitly. The monodomain Eq. (2), the ionic model Cherry et al. (2007), and fiber generation Algorithm 1 were implemented in C++ as part of the cardiac biomechanics finite element library BeatIt^1^. The implementation uses the parallel finite element library libMesh (Kirk et al., 2006), PETSc (Balay et al., 2015), and HYPRE linear solvers (Falgout and Yang, 2002). Visualization and post-processing use Paraview (Ahrens et al., 2005).

## 3 Results

### 3.1 Patient-specific anatomies

**FIGURE 7:**
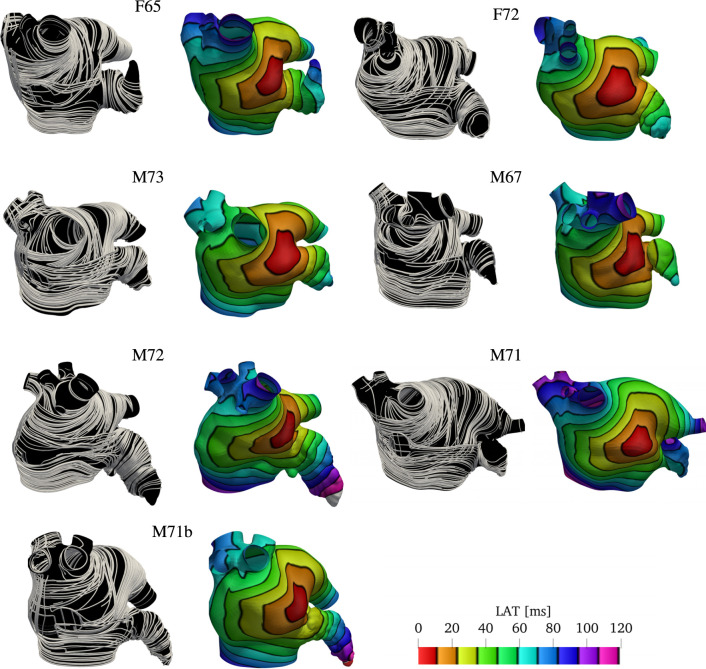
Fibers and local activation times for all geometries other than M75 and M52. Notice we show the final fiber architecture, with endocardial and epicardial layers superposed, in this visualization. Although the size of the geometries look similar, the sizes of the LA are different. That is evident by denser isolines and longer activation times in the LAA and PVS for larger geometries, such as M71.

To show the robustness and flexibility of the proposed algorithm on handling a variety of anatomies, we now use it to assign anatomical regions and fiber orientations in all the remaining patients shown in Figure 1. We follow the same pipeline described in Sections 2.2 and 2.3 to generate physiological models and to simulate the propagation of the electrical stimulus in the LAs. The only changes necessary are in the boundary conditions for *ϕ*_3_ and *ϕ*_4_, and the choices of some values of the threshold parameters *α*_*i*,*k*_ and *β*_*i*,*k*_. The changes in boundary conditions for *ϕ*_3_ and *ϕ*_4_ are done such that the fields capture the expected distances. The changes in thresholds are done such that the anatomical subregions and fiber directions resemble the structures reported in atrial anatomy studies (Ho and Sanchez-Quintana, 2009; Ho et al., 2012; Sánchez-Quintana et al., 2014). More details on the parameter choice are discussed in the supplementary material. The resulting overlapping endocardial and epicardial muscle fiber layers are illustrated in Figure 7, next to the corresponding local activation times (LAT) produced by the numerical solutions to the monodomain model (2).

### 3.2 Comparison with previous rule-based LA fiber models

**FIGURE 8:**
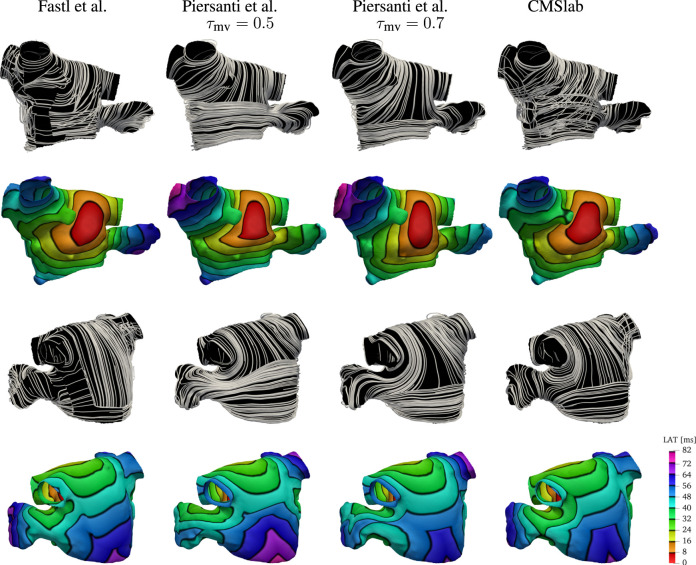
Comparison of the results obtained using the proposed fiber construction algorithm and previously proposed by Fastl et al. (2018) and Piersanti et al. (2021). Using one of the three geometries provided by Fastl et al. (2018), we have built the fibers using the method proposed by Piersanti et al. (2021) using two possible threshold values for the parameter *τ*_mv_ defining the extent of the Bachmann’s bundle. The fiber direction for the Fastl et al. (2018) model construction were available with the mesh. This comparison shows that the proposed algorithm for the definition of the fiber field on the left atrium capture the essential features of the complex algorithm proposed by Fastl et al. (2018), while maintaining the simplicity of the Piersanti et al. (2021). Because the simple algorithm proposed by Piersanti et al. (2021) considers only a single layer of fibers, the activation times on the anterior wall show a higher degree of anisotropy. Note that with this algorithm, the right superior pulmonary vein is one of the last regions to be activated, whereas propagation in the LAA does not follow the expected ostium-to-tip route.

We compare the fiber fields and the activation maps generated using the proposed algorithm with two methods previously published (Fastl et al., 2018; Piersanti et al., 2021). Because the anatomies and fiber fields published in Fastl et al. (2018) are publicly available online^2^, this comparison is based on one of their anatomical models (“03patient”). To generate the fiber architecture using Algorithm 1, we defined seven boundary sets: endocardium; epicardium; mitral valve ring; and four pulmonary veins rims along with two landmark points: the tip of the LAA and the center of the FO. Although we hand-selected the boundary sets, the center of the FO and LAA tip landmarks were chosen by inspecting of the fiber architecture of the original model (Fastl et al., 2018). The same landmark point for the LAA tip was used to reproduce the fiber orientation using the algorithm proposed by Piersanti et al. (2021). For this single layer algorithm, we built two possible variants of the fiber orientation by setting the threshold parameter *τ*_mv_ to 0.5 or to 0.7. As illustrated in Figure 8, changing this parameter changes the size of the mitral valve fiber bundle.

After the fiber fields were defined using the various algorithms, we computed the activation maps by solving the monodomain model with a current stimulus at the center of the anterior wall. Figure 8 shows how the different LA fiber architecture affects the propagation of the electric signal. Comparing the activation times, we see that the signal reached the tip of the LAA at 72 ms in the fiber architecture generated using the algorithm of Fastl et al. (2018), and at 56 and 64 ms using constructions based on the method of Piersanti et al. (2021). In contrast, the proposed algorithm activates the tip of the LAA at 72 ms, as in the construction following Fastl et al. (2018). Moreover, the activation pattern on the LAA is concentric when using both our construction and the construction of Fastl et al. (2018), but this pattern is not observed on either of the constructions following Piersanti et al. (2021). This is because the LAA fibers generated following Piersanti et al. (2021) are not oriented circumferentially with respect to the LAA ostium. Additionally, the propagation of the signal on the anterior wall is more anisotropic using constructions based on Piersanti et al. (2021). This is because our model and the construction following Fastl et al. (2018) have overlapping fiber bundles, whereas the constructions following Piersanti et al. (2021) have only one fiber layer. Finally, in both architectures based on Piersanti et al. (2021), the right PVs took about 8 ms longer to activate than the other two fiber architectures.

### 3.3 Comparison with an atlas-based LA fiber model

We compare the fiber fields and the activation maps generated using the proposed algorithm with the atlas-based method proposed by Roney et al. (2021). Those authors provide^3^ endocardial and epicardial left and right atrial surfaces for each of the seven anatomies included in their study, together with the fiber fields and the universal atrial coordinates (Roney et al., 2019). For this comparison we chose to use the first of their anatomies. Because in this anatomy the endocardial and epicardial surfaces intersect, we compare our model construction directly on the given surfaces. To generate the fiber architecture using Algorithm 1, we defined five boundary sets, the mitral valve ring and four pulmonary veins rims along with two landmark points, the tip of the LAA and the center of the FO. To use Algorithm 1 on a surface, we do not solve for the transmural field *ϕ*_0_ and we directly assign the direction s to the surface normal. Because the surfaces represent either the endocardial or epicardial layers, a variable layer *ℓ* is also provided as input to the algorithm.

**FIGURE 9:**
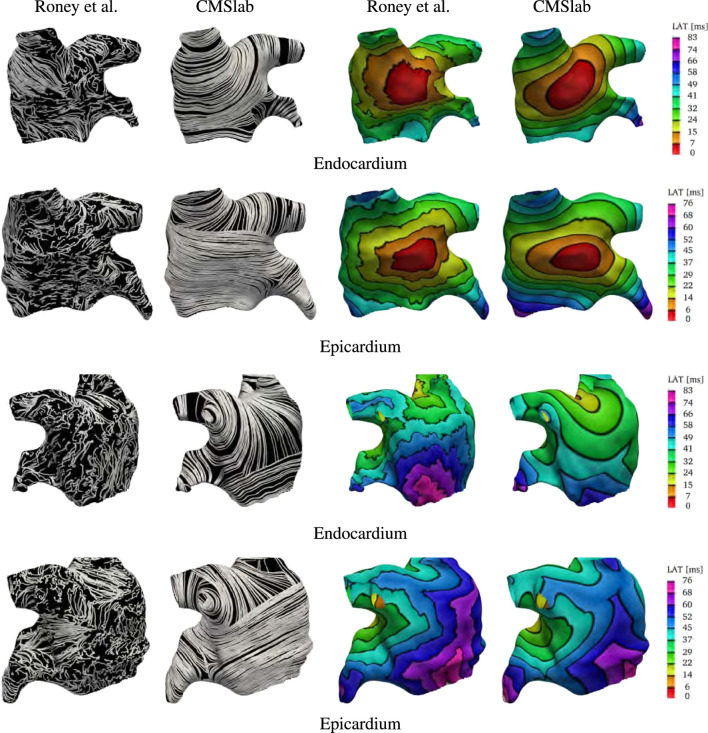
Comparison of the results obtained using the proposed fiber construction algorithm and the atlas based method proposed by Roney et al. (2021). Using the first of the geometries provided by Roney et al. (2021), we built our approximation to the fiber field based on the endocardial and epicardial surfaces. This comparison shows that the major physiological fiber bundles can be captured by our algorithm on any given geometry in contrast to the mapping achieved using the atlas-based method. The resulting fibers generates different activation times, especially on the endocardial surface. Note that the smoothness of the fiber field is reflected in the final activation times.

After the fiber fields were defined using the proposed algorithm, we computed the activation maps by solving the monodomain model with a current stimulus at the center of the anterior wall for both endocardial and epicardial surfaces. Figure 9 compares the fibers and activation maps obtained with the two algorithms. The atlas reconstructed fibers show little organization of the fiber bundles, in contrast with the proposed algorithm. This is reflected in the propagation of the electric signal, where the atlas-based activation maps display a rough wavefront. Comparing the activation times, we see that the signal reached the tip of the LAA at 57 ms (on the endocardium) and 60 ms (on the epicardium) in the fiber architecture generated using the atlas-based algorithm, and at 71 ms (on the endocardium) and 81 ms (on the epicardium) using the constructions based on the proposed method. Note that in the provided surfaces, the wall thickness at the tip of the LAA can be as large as 7 mm. The last regions to be activated were the mitral valve ring at the posterior wall for the atlas-based fibers (84 and 76 ms for the endocardial and epicardial surfaces respectively), and the tip of the LAA with our fiber generation algorithm (73 and 84 ms for the endocardial and epicardial surfaces respectively).

### 3.4 Comparison with rule-based modified dijkstra algorithm

We compare the fiber architecture created by the proposed algorithm with the one created by Wachter et al. (2015) ^4^. The latter approach calculates paths between 22 seed points using a modified version of Dijkstra’s algorithm. We perform the comparison using the example mesh by Loewe et al. (2016) with the seed points selected by Wachter et al. (2015). Because the mesh has some overlapping regions, it is not clear to which extent the algorithm proposed in Wachter et al. (2015) captures the LA fiber architecture. Specifically, the LAA is attached to the left superior PV, which is also attached to the lateral wall.

**FIGURE 10:**
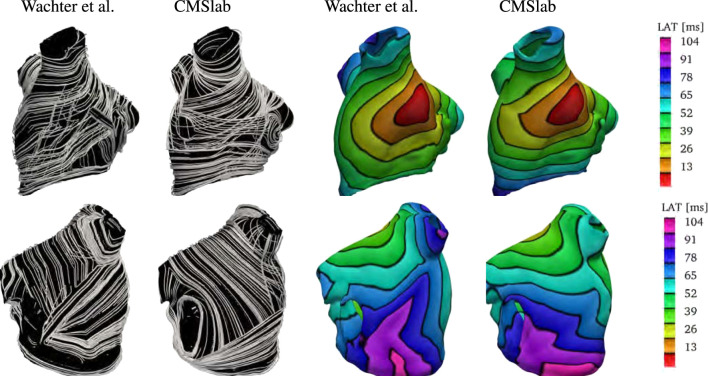
Comparison of the results obtained using the proposed fiber construction algorithm and previously proposed by Wachter et al. (2015). Using the geometry provided by Loewe et al. (2016), we have built our approximation to the left atrial fiber architecture. Notice that the epicardial fiber structure proposed by Wachter et al. (2015) is dominated by the septopulmonary bundle in the anterior wall. The Bachmann’s bundle is defined in a narrower area than in the proposed algorithm. Therefore, the proposed algorithm yields an activation pattern with a pronounced horizontal component when compared to the model created by Wachter et al. (2015). In contrast with the model proposed by Wachter et al. (2015), the proposed algorithm yields longitudinal fibers in the inferior part of the epicardial septal region. This difference results in earlier activation of the left inferior PV in the former model than in the latter one. Besides that, in the model by Wachter et al. (2015), the myocardial strands cross the posterior wall in a higher position than the proposed model. This leads to a more oblique activation pattern in the posterior wall of the former model than in the latter one.

Figure 10 shows the anterior and posterior views of the fiber architectures and activation maps obtained by the proposed algorithm and the one by Wachter et al. (2015). The model by Wachter et al. (2015) presents a narrow Bachmann’s bundle on the anterior wall, which results in an approximately even activation pattern in the anterior and septal regions. In contrast, the proposed algorithm leads to a rather pronounced horizontal activation pattern. Besides that, the proposed algorithm yields longitudinal fibers in the inferior part of the epicardial septal region, whereas the algorithm developed by Wachter et al. (2015) has circular fibers in such region. This difference results in earlier activation of the left inferior PV in the former model than in the latter one. Besides that, in the model by Wachter et al. (2015), the myocardial strands cross the posterior wall in a higher position than in the proposed model. This leads to a more oblique activation pattern on the posterior wall when compared to the proposed model.

Finally, we remark that it is unclear how this method performs in geometries with PV anomalies.

## 4 Discussion

While computed tomography is an essential component of peri-procedural planning of WATCHMAN device placement to occlude the left atrial appendage Kaafarani et al. (2020), the collected images do not capture the right atrium adequately. For this reason, we limited our study to the reconstruction of the fibers in the left atrium. The fiber architecture of the left atrium is complex and difficult to quantify. Anatomical studies have revealed that the left atrium is composed of overlapping fiber bundles crossing each other at various angles depending on the location (Ho and Sanchez-Quintana, 2009; Ho et al., 2012; Sánchez-Quintana et al., 2014; Pashakhanloo et al., 2016). Structural remodeling of the left atrium and fibrosis associated with atrial fibrillation can lead to local changes of the fiber structure via deposition of interstitial collagen and remodeling of the extracellular matrix (Al Ghamdi and Hassan, 2009; Jansen et al., 2020).

Based on the observation made in the studies by Pashakhanloo et al. (2016), we use the following simplifying assumptions to build our model. In the fossa ovalis we assume the fibers run longitudinally in the epicardial layer, and circumferentially in the endocardial layer. The fibers inferior to the fossa ovalis are defined to run circumferentially and extend to the posterior and anterior walls, The fibers in the lateral wall run circumferentially. In the roof, a group of longitudinal fibers transition to a circular pattern encircling the four pulmonary veins. In the lateral wall and in the roof, the fibers have unimodal transmural fiber distribution. In the pulmonary veins, the fiber are circumferential in both endocardial and epicardial layers, except for the epicardial layer of the right inferior pulmonary vein where they run longitudinally. The latter pattern, also defined in the epicardial layer of inferior part of the septum, may be able to account for the obliquely running fibers originated between the right pulmonary veins incorporated into the septal wall. The fibers in the anterior wall descend obliquely into the bottom part of septal wall near the mitral valve ring. These fiber bundles are extensions of the fiber tracts from the roof and lateral wall. The Bachmann’s bundle is the main muscle bundle running in the epicardial layer of the anterior wall. Its extension on the left side of the LA was defined such that it splits into 2 bands encircling the left atrial appendage (Cabrera et al., 2008; Ho et al., 2012; Sánchez-Quintana et al., 2014; Pashakhanloo et al., 2016). The circular pattern extends around and up to the tip of the appendage. The main muscle bundle in the posterior wall is the septopulmonary bundle, running in the anterior-to-posterior direction and crossing with the circumferential fibers coming from the lateral wall.

Many computational studies on the left atrium relied on the local fiber direction (Dössel et al., 2012; Krueger et al., 2014; McDowell et al., 2015; Rossi et al., 2018). Although a variety of approaches to assign the muscle orientation in the atria have been proposed (Hermosillo, 2008; Krueger et al., 2011; Labarthe et al., 2012; McDowell et al., 2012; Satriano et al., 2013; Labarthe et al., 2014; Fastl et al., 2018; Roney et al., 2018, 2021; Saliani et al., 2019; Piersanti et al., 2021), many of them are challenging to implement. Further, when a new algorithm is proposed, it is rarely compared against prevoius approaches. This study presents the first comparison between three rule-based algorithms for generating atrial fibers on a patient-specific anatomy.

We considered two methods (Fastl et al., 2018; Piersanti et al., 2021) in which the local variation of the fiber direction in the left atrium is described by harmonic fields. The method developed by Fastl et al. (2018) requires the prescription of 184 landmark points, along with 272 auxiliary lines that subdivide the endocardial and epicardial surfaces in 151 regions. By solving the Laplace equation in each of these regions, that method defines various harmonic fields whose gradients correspond to the local fiber direction. Once the fiber fields are defined on the epicardial and endocardial surfaces, they can be extended in the interior of the left atrium using interpolation. Prior work investigated the influence on local activation times of four different transmural interpolations and noted only minor differences between them. This suggests a negligible effect of the transmural fiber distribution, supporting the idea that a two-layer description of the muscle bundles provides a sufficient approximation of the left atrial anisotropy. However, the study of Fastl et al. (2018) only compared that fiber generation method to activation maps obtained from isotropic models. Unfortunately, this comparison does not give any insight on whether that algorithm provides a better approximation of the fiber direction with respect to other methods. Fastl et al. (2018) states that their method reduces the intra- and interobserver variability while increasing reproducibility for large patient cohorts. Unfortunately, the complexity of the algorithm, with automated transfer of predefined landmarks from an average atrial geometry to a personalized atrial geometry and *a priori* definition of rules to generate fibers, prevents its widespread use. Nonetheless, because the authors have published the anatomies and the corresponding fiber fields, it is possible to compare directly the output of their algorithm with the one detailed herein.

A much simpler algorithm was developed by Piersanti et al. (2021) to generate the fiber fields in the left atrium. Although their algorithm extends to the creation of the fibers in the whole heart, here we will discuss only the portion of the algorithm that refers to the atrium. In their construction, four harmonic fields are defined on the volumetric representation of the left atrium. Using threshold on the harmonic fields and their gradients, they subdivide the atrium in three regions and reconstruct the local fiber directions. This method is simple to understand and implement, but it does not capture some of the important characteristics of atrial fibers architecture: the muscle fibers in the left and right carina are not correctly reproduced; the size of the Bachmann’s bundle can be controlled only at the expense of the left atrial appendage fibers; and the method does not provide a way to separate endocardial and epicardial muscle bundles. In our electrophysiology simulations using the Piersanti et al. (2021) fiber fields, the lack of multiple layers of fibers resulted in more pronounced anisotropy in regions where the two-layer model of Fastl et al. (2018) finds a more isotropic behavior due the different alignments of fibers in the different layers.

Here, we propose a new algorithm to generate the fiber orientation on the left atrium capable of reproducing activation times in electrophysiological simulations similar to the complex algorithm proposed by Fastl et al. (2018), that maintains the simplicity of the method by Piersanti et al. (2021). To achieve a good agreement between the anatomical fiber orientation and the model we compute seven harmonic fields. Each of these fields can be thought of a normalized distance between specific atrial dimensions. Specifically, the fields represent the transmural, left-to-right, anterior-to-posterior, and superior-to-inferior distances. Some fields can be interpreted as distances connecting multiple regions of the left atrium. The solution to these fields can be computed using standard numerical methods for partial differential equations. Using these seven harmonic fields and their gradients, the proposed algorithm generates successive endocardial and epicardial subdomains in which the fiber orientation is assigned. The decomposition of the left atrium in only 17 regions is a substantial simplification over the algorithm of Fastl et al. (2018). Additionally, comparing the fiber structure defined by our algorithm with that of Fastl et al. (2018) in one of their geometries, we found our algorithm to better represent the fiber orientation in the left lateral ridge.

Similarly to the approach by Fastl et al. (2018), our algorithm can be modified to capture different transmural variation in the fiber directions. Because the analysis of Fastl et al. (2018) found only minor differences between using two or more than two overlapping layers, we built our algorithm considering endocarial and epicardial layers only with a sharp transition in the midwall. The definition of the endocardial and epicardial layers is an improvement over the algorithm proposed by Piersanti et al. (2021): comparing electrophysiological activation times obtained using the various models, in which an initial stimulus on the anterior wall, at the top of the Bachmann’s bundle, the propagation towards the mitral valve ring is faster in models that include multiple layers of fibers.

We further extended our analysis by comparing the proposed algorithm with the rule-based model introduced in Wachter et al. (2015), which is based on a modification of Dijkstra’s algorithm. The comparison revealed that the algorithm by Wachter et al. (2015) yields a narrower Bachmann’s bundle, and taller horizontal and oblique myocardial strands on the posterior wall when compared to the proposed model. Besides that, the fibers defined by Wachter et al. (2015) are defined horizontally in the septum, which contrasts with the longitudinal epicardial fiber direction in the inferior part of the septum in the proposed algorithm. These differences impacted the overall activation map pattern, being mostly noticeable in the inferior pulmonary vein and posterior wall.

We remark, however, that the proposed algorithm does not give a quality fiber field in the geometry provided by Loewe et al. (2016) since the left atrial geometry is not properly pre-processed. Indeed, such geometry has overlapping structures that are separated such as the left atrial appendage and the left superior pulmonary vein, or the left inferior pulmonary vein and the left lateral wall. Besides that, at this point, we are unsure on whether this method is extendable to anatomies with different pulmonary anomalies.

We also have compared our model construction to an atlas-based algorithm (Roney et al., 2021). Using *ex vivo* information on the atrial fibers, Roney et al. (2021) created a reference atlas fiber field. By means of universal atrial coordinates (Roney et al., 2019), the reference fibers are mapped on a square representing the left atrial geometry. Defining the universal atrial coordinates on a new left atrium, it is straightforward to map the reference fibers from the atlas back to reconstruct the local muscle bundle orientation. For this comparison, we used one of their available anatomies. Because in this anatomy the endocardial and epicardial surfaces intersect, we compared our model construction on the given surfaces. The atlas reconstructed fibers showed little organization of the fiber bundles, which resulted in a rough wavefront in comparison with the results obtained by the proposed algorithm. Although this approach seems reasonable, at this time, the large variation in left atrial and left atrial appendage morphologies seems to limit its applicability. Additionally, it is unclear if this algorithm can be easily extended to anatomical variations of the left atrium, and the behavior of such fiber reconstruction algorithm under these circumstances remains unclear.

The approaches discussed previously define the fiber orientation on the most common phenotype of the left atrium with four pulmonary veins. It is unclear how to extend them to the large variety of common configurations of the left atrium and pulmonary veins (Chen et al., 2017). In some cases, smaller pulmonary veins are just capped and treated as part of the left atrium. This was the case for left atrial geometry we used to compare between the methods (Figure 8), published by Fastl et al. (2018). In the cohort we studied, most of the patients have some variations of the pulmonary veins: two out of nine patients had a common left pulmonary trunk, and all patients had one or more right accessory pulmonary veins. We have demonstrated that the proposed algorithm can handle all these variants. This was achieved by changing the boundary conditions for two of the harmonic fields. Specifically, for the anterior-posterior (*ϕ*_3_) and inferior-superior (*ϕ*_4_) distance fields, we set intermediate values for the boundary conditions on the accessory right pulmonary veins. On the common left pulmonary trunk we impose free conditions (homogeneous natural/Neumann) when computing the anterior-posterior harmonic field (*ϕ*_3_). To our knowledge, the proposed algorithm is the first to account for these variations in the left atrial anatomies.

We demonstrated the robustness of our approach using nine anatomies of the left atrium obtained from patients undergoing WATCHMAN device implantation. These patients encompass a large variety of morphology variants, both in terms of left atrial appendages and pulmonary veins configurations. The proposed algorithm was able to define approximate fiber fields on all these patients while keeping the same overall structure of the muscle bundles. Using the monodomain model to evaluate activation times when a stimulus is applied on the anterior wall, at the edge of the Bachmann’s bundle, to replicate normal activation, we have shown (Figure 7) that in all the anatomies we recognize mainly two activation pathways: one circumferential following the Bachmann’s bundle and one longitudinal following the septoatrial bundle. Even though we used fixed values of *σ* ^f^, *σ* ^s^, and *σ* ^n^ across the left atrium, the conductivity tensor accounted for changes in the local frame of reference and was expressed as ***σ*** = *σ* ^f^ ***f*** ⊗ ***f*** + *σ* ^s^ ***s*** ⊗ ***s*** + *σ* ^n^ ***n*** ⊗ ***n***.

In conclusion, we presented a new algorithm that balances algorithmic complexity and physiological detail for determining the muscle fiber architecture of the left atrium. The algorithm is robust and flexible, and it can be applied to many morphological variations of the left atrium. Although atrial fibrillation patients may have structural remodeling, which can completely alter the local substrate and conduction properties, the proposed algorithm is relatively simple while yielding results that are similar to a much more complex construction approach. If information about local tissue fibrosis is available this can be incorporated in the physical models without necessarily altering the baseline fiber orientation.

## Data Availability

Some datasets presented in this article are not readily available because of patient confidentiality. Requests to access those datasets should be directed to the authors. All other raw data supporting the conclusions of this article data will be made available by the authors.

## Footnotes

1 Available at https://github.com/rossisimone/beatit/tree/master/examples/example_AFib_fibers.

2
http://doi.org/doi:10.18742/RDM01-289

3
https://doi.org/10.5281/zenodo.3764916

4 code available at https://github.com/KIT-IBT/RESILIENT
