# Supplementary material for: Rule-based definition of muscle bundles in patient-specific models of the left atrium
Source: Front Physiol. 2022 Oct 12;13:912947. doi: 10.3389/fphys.2022.912947 (PMC9597256; doi:10.3389/fphys.2022.912947)
Supplement: Supplementary file 2 [file DataSheet3.pdf]

frontiers

Supplementary Material

1 SUPPLEMENTARY DATA

1.1 Patients' threshold parameters

	F65	F72	M52	M67	M71	M71b	M72	M73	M75
$\alpha_{\text{ENDO-EPI}}$	0.5	0.5	0.5	0.5	0.5	0.5	0.5	0.5	0.5
α_{AF}	0.85	0.85	0.85	0.85	0.85	0.85	0.85	0.85	0.85
α_{PF}	0.65	0.65	0.65	0.65	0.65	0.65	0.65	0.65	0.65
α_{RA}	0.78	0.67	0.75	0.69	0.75	0.83	0.78	0.78	0.78
α_{AP}	0.475	0.43	0.525	0.45	0.45	0.5	0.5	0.5	0.52
α_{RIPV}	0.35	0.3	0.3	0.33	0.34	0.35	0.36	0.3	0.36
α_{LR}	0.58	0.45	0.65	0.5	0.53	0.71	0.5	0.65	0.52
α_{LA}	0.29	0.12	0.3	0.16	0.3	0.32	0.11	0.27	0.19
α_{BB}	0.45	0.45	0.48	0.49	0.48	0.48	0.35	0.4	0.45
α_{LLR}	0.125	0.12	0.15	0.1	0.14	0.2	0.13	0.2	0.13
α_{AL}	0.5	0.5	0.55	0.55	0.55	0.6	0.6	0.5	0.6
α_{AR}	0.499	0.499	0.499	0.499	0.499	0.499	0.499	0.499	0.499
α_{EL}	0.1	0.04	0.1	0.1	0.499	0.1	0.1	0.1	0.06
α_{LAL}	0.48	0.45	0.48	0.4	0.46	0.49	0.42	0.48	0.4
α_{LE}	0.51	0.45	0.49	0.3	0.4	0.0	0.45	0.45	0.45
LAA x	-3.59	-1.24	-4.775	-2.537	-2.133	-5.247	-4.084	-4.16	-3.95
LAA y	3.07	5.27	3.39	3.933	4.836	3.597	6.487	3.27	4.03
LAA z	5.47	2.29	1.197	1.793	4.850	-0.614	-0.284	1.29	1.12
LAA r	0.0125	0.0125	0.0125	0.0125	0.0125	0.0125	0.0125	0.0125	0.0125
FO x	3.06	4.2	3.829	3.215	4.302	2.217	3.575	2.71	2.7
FO y	2.26	0.55	0.866	0.044	2.105	2.143	2.027	1.32	1.6
FO z	3.70	2.54	4.28	3.272	4.514	3.713	3.258	2.56	2.1
FO r	0.02	0.02	0.02	0.02	0.02	0.02	0.02	0.02	0.02
α_{FO}	0.448	0.275	0.3325	0.24	0.253	0.35	0.35	0.4	0.29
α_{S}	0.469	0.308	0.355	0.2615	0.269	0.374	0.373	0.424	0.317
α_{L}	0.487	0.332	0.375	0.2275	0.2825	0.388	0.388	0.45	0.34
α_{LAA}	0.505	0.365	0.395	0.307	0.3	0.4325	0.415	0.475	0.37

Table S1. CMSlab threshold parameters on various morphologies.

1.2 Comparison threshold parameters

	03 Patient
$\alpha_{\text{ENDO-EPI}}$	0.5
α_{AF}	0.85
α_{PF}	0.65
α_{RA}	0.86
α_{AP}	0.385
α_{RIPV}	0.25
α_{LR}	0.45
α_{LA}	0.16
α_{BB}	0.4
α_{LLR}	0.08
α_{AL}	0.5
α_{AR}	0.499
α_{EL}	0.08
α_{LAL}	0.47
α_{LE}	0.48
LAA x	8.61
LAA y	1.91
LAA z	4.45
LAA r	0.005
FO x	2.53
FO y	1.41
FO z	2.35
FO r	0.005
α_{FO}	0.422
α_{S}	0.439
α_{L}	0.485
α_{LAA}	0.515

Table S2. CMSlab threshold parameters on Fastl et al. 03 patient.

2 BOUNDARY CONDITIONS AND SOLUTIONS FOR THE FIELDS ϕ_3 AND ϕ_4 FOR ALL ANATOMIES

The boundary conditions for ϕ_3 are chosen such that the anterior-posterior threshold α_{AP} is about 0.5. This ensures that the limit of the anterior wall is half-way between the right and left pulmonary veins. The boundary conditions for ϕ_4 are such that the outermost pulmonary veins are assigned to $g = 0$, and the inner pulmonary veins are assigned $g = 0.1$. This choice is made so that this field can be used to define the epicardial fibers on the right inferior pulmonary veins and on the right antra. In addition, the fossa ovalis and the left atrial appendage are set to 0.5, and the mitral valve ring is assigned to 1. This second piece is important when defining the Bachmann's bundle.

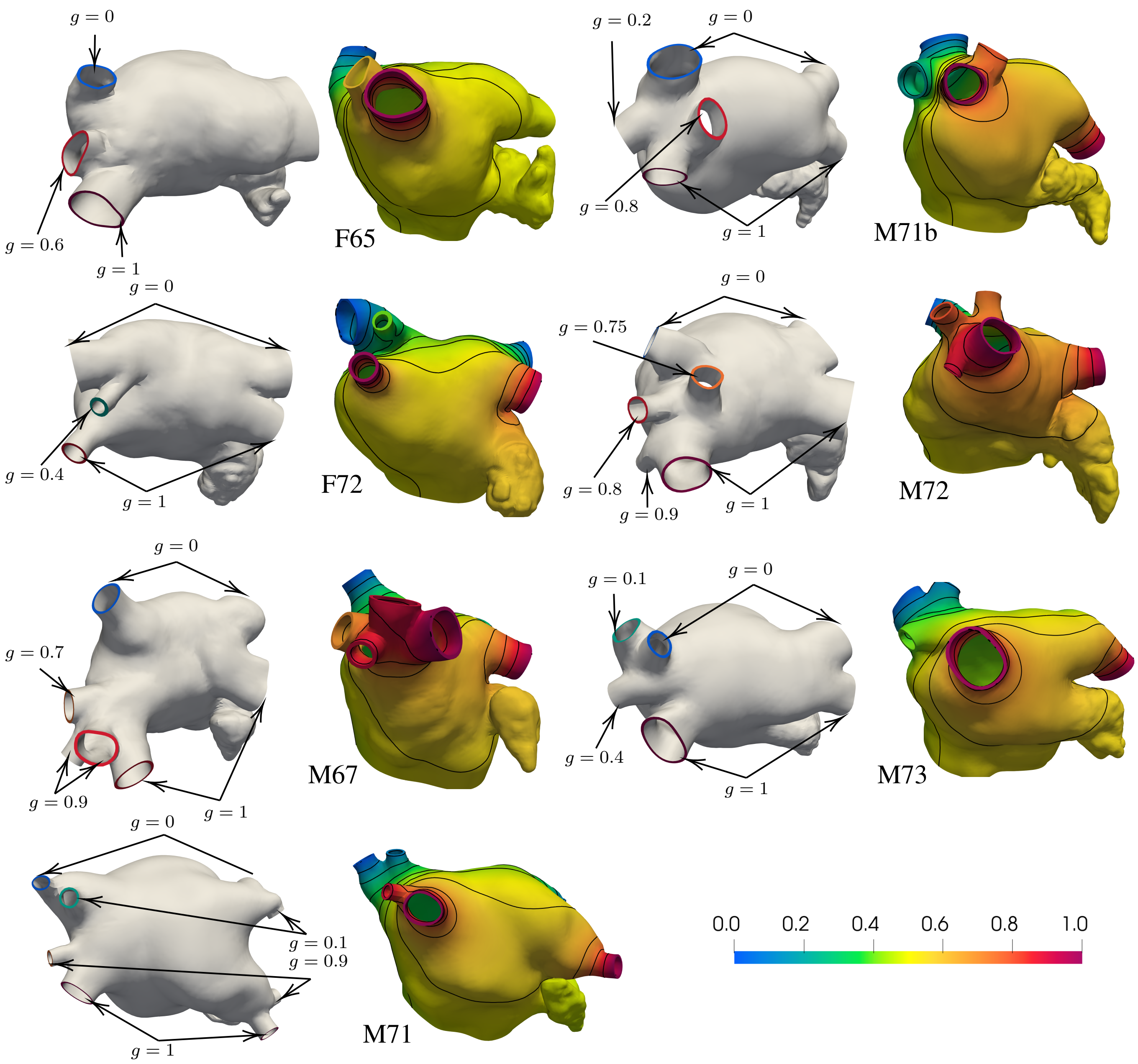

Figure S1. Boundary data and harmonic field ϕ_3 on all geometries other than M75 and M52. We set $g = 0$ on the right inferior pulmonary veins and $g = 1$ right superior pulmonary veins. The g values on the accessory pulmonary veins are set to intermediate values between 0 and 1. On the F65 left common pulmonary trunk, we impose natural boundary conditions.

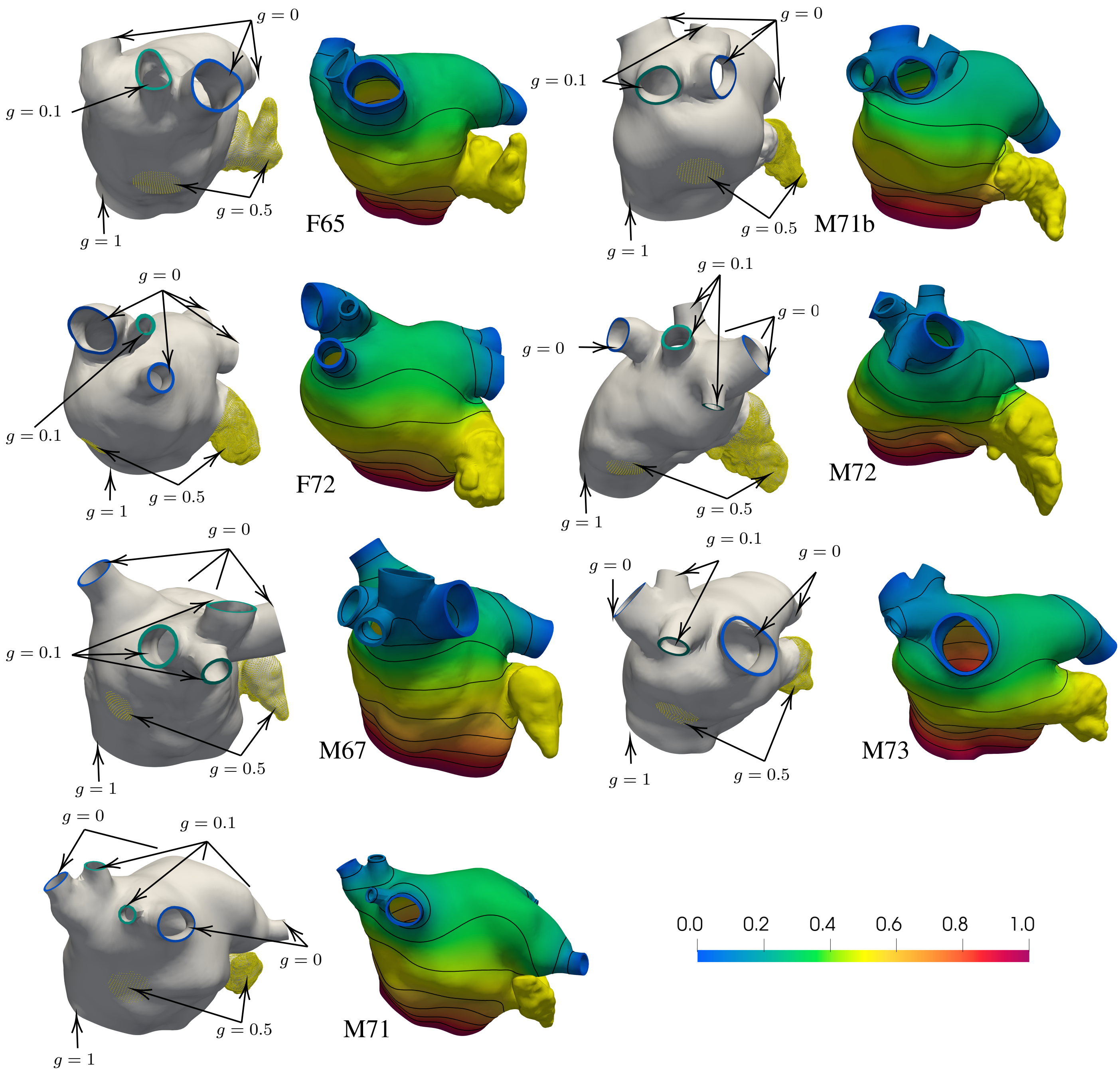

Figure S2. Boundary conditions and harmonic field ϕ_4 for all geometries other than M75 and M52. The boundary conditions are such that the outermost right pulmonary veins are assigned to $g = 0$ and the innermost ones are assigned to $g = 0.1$. Note that for anatomy M71, which has four left pulmonary veins, the innermost boundary conditions follow the same pattern as in the right pulmonary veins. For all the remaining geometries, all the left pulmonary veins are assigned to $g = 0$. The LAA and FO are assigned to $g = 0.5$ and the MV ring is assigned to $g = 1$.

3 PARAMETERS USED TO REPRODUCE THE PIERSANTI ET. AL (2021) MODEL

To reproduce the left atrial fiber architecture model by Piersanti et al. (2021) on "03patient" [1], we solved four Laplace equations for $\tilde{\phi}_i$, $i = \{0, \ldots, 3\}$. For the field $\tilde{\phi}_0$, we set $g = 0$ on the endocardium and $g = 1$ on the epicardium. For field $\tilde{\phi}_1$, the right pulmonary veins are set to 2, the left pulmonary vein is set to 0, the mitral valve ring is set to 1, and the appendage tip is set to -1. The boundary conditions for field $\tilde{\phi}_2$ is 0 on the right pulmonary veins and 1 on the left pulmonary veins. Finally, for the field $\tilde{\phi}_3$, the mitral valve ring is set to 1, and the pulmonary veins and appendage tip are set to 0. Then, to create the left atrial fiber architecture, we implemented the **Algorithm 2** using thresholds $\tau_{\text{lpv}} = 0.85$ and $\tau_{\text{rpv}} = 0.1$. In the two simulations presented in the results section, the parameter τ_{mv} is set to either 0.5 or 0.7.

Algorithm 2: Left atrial fiber architecture by Piersanti et al. (2021).

Given the solutions $\tilde{\phi}_i$, with $i = 0, 1, 2, 3$;
if $\tilde{\phi}_3 > \tau_{mv}$ **then** $\boldsymbol{f} = \nabla\tilde{\phi}_3 \rightarrow$ MV ;
else
    if $\tilde{\phi}_2 \geq \tau_{lpv}$ *or* $\tilde{\phi}_2 \leq \tau_{rpv}$ **then** $\boldsymbol{f} = \nabla\tilde{\phi}_2 \rightarrow$ LPV and RPV ;
    else $\boldsymbol{f} = \nabla\tilde{\phi}_1 \rightarrow$ LAS, LSW, LAW, LAR and LAA.;

MV – mitral valve, LPV – left pulmonary vein, LRV – right pulmonary vein, LAS – right and left atrial septum, LSW – right and left atrial lateral aall , LAW–left septum wall, LAR– left atrial roof.

REFERENCES

Cabrera, J. A., Ho, S. Y., Climent, V., and Sanchez-Quintana, D. (2008). The architecture of the left lateral atrial wall: a particular anatomic region with implications for ablation of atrial fibrillation. Eur. Heart J. 29, 356–362

Fastl, T. E., Tobon-Gomez, C., Crozier, A., Whitaker, J., Rajani, R., McCarthy, K. P., et al. (2018). Personalized computational modeling of left atrial geometry and transmural myofiber architecture. *Med Image Anal.* 47, 180–190

Ho, S. Y., Cabrera, J. A., and Sanchez-Quintana, D. (2012). Left atrial anatomy revisited. *Circ Arrhythm Electrophysiol.* 5, 220–228

Piersanti, R., Africa, P. C., Fedele, M., Vergara, C., Dedè, L., Corno, A. F., et al. (2021). Modeling cardiac muscle fibers in ventricular and atrial electrophysiology simulations. *Comput Methods Appl Mech Eng.* 373, 113468

Sánchez-Quintana, D., López-Mínguez, J. R., Macías, Y., Cabrera, J. A., and Saremi, F. (2014). Left atrial anatomy relevant to catheter ablation. *Cardiol. Res. Pract.* 2014

[1] Available at `http://doi.org/doi:10.18742/RDM01-289`.
